# Our youth, our compass: families walking with younger‐onset dementia shine light on stigma

**DOI:** 10.1002/alz.089157

**Published:** 2025-01-09

**Authors:** Diana Shulla Cose, Stephanie Fitzgerald, Patti LaFleur, Bree Ruge

**Affiliations:** ^1^ Lorenzo’s House, Chicago, IL USA

## Abstract

**Purpose:**

For youth walking with a parent’s younger‐onset dementia diagnosis, our voices are the softest, yet our journey is often the hardest. We are helping to open the shades and bring light. We are telling the stories of our Mother’s and our Father’s. We are the survivors who choose to advocate for a more just world for our families. Through the EmpowermentPLUS Model, we are bringing strength to stigma.

**Project Plan:**

The three components of the EmpowermentPLUS Model include (1) recognize, (2) respond, and (3) empowerx2. For example, when I feel I am experiencing stigma as a daughter of my parent living with dementia–at a family party, grocery store, park or hospital, I am (A) able to recognize it, and (B) I have the tools to respond, rather than responding with fight, flight, or freeze and (C) the last piece is that my mindful and informed response empowers me as a daughter, and the PLUS symbolizes that the person who triggered the stigma is also empowered by being educated.

**Outcomes:**

During our international Youth Summit, with support from Alzheimer’s Society of Canada, Alzheimer’s Disease International and London School of Economics, nearly 100 youth worldwide shared personal stories of when they experienced stigma as a younger family walking with dementia, and we taught the EmpowermentPLUS Model as a way to grapple and dismantle it.

**Conclusion:**

We are building our alliance of empowered young ambassadors, and expect more than 200 youth at Lorenzo’s Youth Summit 2024. Our Founding Executive Director, Diana Shulla Cose, and another member of our Lorenzo’s House community will share the youth voices and details of Lorenzo’s House strength to stigma work.

